# The value of including families in the treatment of anorexia nervosa

**DOI:** 10.1002/erv.2816

**Published:** 2020-12-22

**Authors:** Janet Treasure, Stacey Parker, Oyenike Oyeleye, Amy Harrison

**Affiliations:** ^1^ Section of Eating Disorders Department of Psychological Medicine King's College London Institute of Psychiatry, Psychology and Neuroscience London UK; ^2^ South London and Maudsley NHS Foundation Trust Adult Inpatient Eating Disorders Service Tyson West 2, Bethlem Royal Hospital Beckenham UK; ^3^ Department of Psychology and Human Development University College London Institute of Education London UK

**Keywords:** anorexia nervosa, eating disorders, family therapy, Maudsley model, systemic therapy

## Abstract

The aim of this paper is to consider family and wider carer involvement in the treatment of anorexia nervosa, and how this can be used to add value to services. We discuss widely adopted interventions involving the family that have been manualised and studied in trials that have outcome measures that are of relevance to illness costs. The therapeutic targets of these interventions range from a focus on feeding to the wellbeing of the whole family. The theoretical models that underpin interventions involving the family/wider carers include both intra and interpersonal processes, with the exception of family‐based therapy, which in its original form holds an agnostic stance towards aetiology. Although formal evaluation of the cost effectiveness of these interventions is minimal, there is evidence that involving the family can reduce bed use and improve the wellbeing of both patients and family members. Moreover, for the most part, these interventions are acceptable to patients and carers. Finally, we consider how these approaches can be disseminated and scaled up more widely into services.

## Highlights

- Several interventions for anorexia nervosa describe the involvement of families in treatment
- These interventions are acceptable to both patients and family members, reduce bed use and improve wellbeing in family members
- These approaches can be scaled up more widely into services

**Abbreviations**
AN: anorexia nervosa
CBT: cognitive behavioural therapy
FBT: family‐based therapy
NMCC: New Maudsley Collaborative Care

## 1 INTRODUCTION

The clinical features of anorexia nervosa (AN) such as the median age of onset at 15 years (Micali et al., 2013), the average illness duration of 10 years, and the stunting of physical and psychological development as a consequence of starvation underline the need to consider how the transition from care within the family of birth to the family of choice may be derailed, warranting attention across the course of the illness. For example, an individual with a pre‐pubertal illness resistant to treatment and managed in child and adolescent services may be fast forwarded into a service that expects adult level autonomy with disconnection from the family just because the eighteenth birthday or some arbitrary marker has been passed (Winston et al., 2012). This disconnect between chronological and developmental age needs to be considered when formulating a case and developing a plan of treatment. Parents are usually intimately involved when the illness develops in childhood and early adolescence. Later, a wider social network including siblings, partners or friends/colleagues may also be needed to bridge developmental gaps. Sadly, social support (e.g. practical and emotional help and care from others) often diminishes over time, leaving patients isolated within the illness (McKnight & Boughton, 2009). Many patients remain financially and socially dependent on their parents or the state over their life time (Hjern et al., 2006). In addition to age, duration and timing of the illness, other facets such as severity and comorbidity (in particular, autistic spectrum disorder (ASD) traits) impact on social connections. Social emotional functioning and its impact on relationships are recognised to be both a risk and maintaining factor for AN (Cardi, Turton, et al., 2017; Treasure et al., 2020). Therefore, targeting social and family functioning is important throughout the course of the illness. This paper aims to discuss why family involvement is important in treatment for AN, discusses examples of how the family can be involved in treatments for AN, possible benefits in terms of resource use and symptom outcomes and what research might be needed next to advance this field.

### 1.1 The impact of anorexia nervosa on families and the social network

The diverse presentation of AN (child/adult onset, acute/chronic, severe/mild malnourishment, comorbid/simple) mean that the use of the term ‘carer’ can be problematic. This term is used in this paper in its broad political sense in order to tap into the policy and resource that may be available. It may include friends, as well as family, or even draw on a compassionate part of the self for those who have become isolated. Eating disorder symptoms are visible and disruptive, and so, the impact on close others is profound. Systematic reviews of carer functioning report high levels of burden which can be associated with anxiety and depression (Anastasiadou, Medina‐Pradas, Sepulveda, & Treasure, 2014). These stressors can impact on interpersonal relationships, leading to high levels of expressed emotion (overprotection, criticism and hostility and lower levels of warmth), accommodation to the illness because of anxiety in the source of support and fragmentation of support (Treasure & Nazar, 2016).

Close others can inadvertently accommodate the illness and be drawn into avoidance and safety behaviours such as reassurance seeking and calibration with others which allow the illness to take a tighter hold. This adaptation may manifest around food and eating, exercise or social behaviours (Treasure & Nazar, 2016; Treasure et al., 2020). In addition, carers can be drawn into enabling illness habits, such as providing money for binge food or ameliorating negative consequences in the bathroom and kitchen. Preliminary surveys to establish the requirements of carers highlight their need for information about the illness and guidelines about how to help (Haigh & Treasure, 2003; Mitrofan et al., 2019; Whitney et al., 2005).

### 1.2 What treatment models have been developed to help with these challenges?

In the following section, we sketch out the underpinning models for family involvement for those interventions that describe how theory or strategy is put into action explicitly in treatment manuals (we are aware that other methods of working on social connection have been developed, but manuals are not yet publicly available). Interventions involving adolescents usually have a focus on the outcomes of the individual patients, whereas treatments delivered to those with a more chronic or adult presentation may consider the broader social impact. All except for family‐based therapy (FBT) have a model which suggests that families can maximise their effectiveness in providing support towards recovery through optimising interpersonal relationships.

## 2 SYSTEMIC FAMILY THERAPY

The model in systemic family therapy is that the problem is not located in the individual themselves but instead lies within interpersonal relationships, social interactions and narratives about the problem. There is no specific emphasis on regular eating or weight restoration, but if these are problems that families bring to sessions, then the therapist will work with them to identify strengths and resources within the family to help them to find new solutions to these difficulties. Manuals are available (Pote, Stratton, Cottrell, Boston, & Shapiro, 2001, Pote, Stratton, Cottrell, Shapiro, & Boston, 2003).

### 2.1 Family‐based therapy also known as the Maudsley Model of family therapy

FBT is a form of systemic family therapy established at the Maudsley Hospital and used as one of the arms of therapy given post‐discharge from inpatient care in a seminal randomised controlled trial (Eisler et al., 1987; Russell Szmukler, Dare, & Eisler, 1987). It was later adapted and developed as the primary treatment for first episode adolescent AN. FBT, in its manualised form, takes an atheoretical stance as a means of presenting a non‐blaming attitude towards families (Lock & Le Grange, 2015). However, some later derivations have been modified to encompass the idea that families may become organised around the illness (Eisler, 2005), and a formulation is used with families. FBT is problem focused, and the treatment targets are clearly re‐establishing regular eating, weight restoration and the reduction of illness behaviours like purging. In the original form, this treatment consists of three phases. Phase one mobilises parents to take the lead on weight restoration. Phase two focuses on transitioning control over food back to the individual at an age‐appropriate level. Phase three focuses on other issues related to typical adolescent development. Other versions of this treatment have been developed whereby the parents and child are predominately seen separately (Le Grange Eisler, Dare, & Russell, 1992), Intensive parent coaching is provided to improve non‐response identified at session 4 of treatment (L'Insalata et al., 2020), or groups of family members are seen together (multifamily therapy; Eisler, 2005). Also, versions have been developed for emerging adults (Dimitropoulos, Farquhar, Freeman, Colton, & Olmsted, 2015).

These family‐based approaches can be contrasted with the approach taken in Cognitive Behavioural Therapy (CBT) which has been used to treat adolescent AN (Dalle Grave et al., 2019) and to view the source of the problem as being within the individual (e.g. the over evaluation of eating, shape and weight and their control). CBT focuses on encouraging the individual to understand and manage their eating disorder behaviours (Dalle Grave et al., 2019). In contrast to FBT, where the family is actively involved, CBT views the family role as useful but not crucial and suggests parents may be involved in treatment and could, for example, be helpful in supporting the individual to implement new behaviours (Fairburn et al., 1999) For adolescents, a three component ‘CBT oriented family module’ is provided within treatment where families are provided with a mixture of educational sessions. These involve understanding the CBT formulation of the eating disorder, supporting with assisting the individual with eating during meal times as a family and creating the optimal family environment to support the patient's efforts to change (Fairburn, 2008).

## 3 THE NEW MAUDSLEY MODEL FOR COLLABORATIVE CARE

New Maudsley Collaborative Care (NMCC) was developed for carers of adults and those with treatment resistant AN. The cognitive interpersonal model upon which this is based describes intra and interpersonal maintaining factors (Schmidt & Treasure, 2006; Treasure & Schmidt, 2013; Treasure & Nazar, 2016; Treasure et al., 2020). These are targeted in treatment with the aim of bridging isolation and fostering healthy connections (Treasure et al., 2015). A shortened form of the intervention is encompassed within the Maudsley Model of outpatient treatment (MANTRA) (one of the treatments of AN recommended by NICE 2017 for adult outpatient care) and in TRIANGLE (Cardi, Ambwani, et al., 2017) and its precursors to augment inpatient care (Hibbs et al., 2015; Magill et al., 2016) or adolescent outpatient care (Hodsoll et al., 2017).

Within NMCC, the definition of carer is not limited to parents, but broadened to include any suitable source of support, such as a friend, sibling, partner or child. NMCC utilises a flexible format to equip carers with skills to not only care for the individual but also to manage their own response to the disorder. This includes building resilience, communication and emotion regulation skills, and addressing accommodation and enabling behaviours and the tendency for fragmentation within the support network (Treasure & Nazar, 2016). The first phase of treatment is to share state‐of‐the‐art understanding of the risk and maintaining factors of the illness, in particular, those that impact on social functioning and eating regulation. Phase 2 involves a consideration of how carers themselves may have become entangled with the illness possibly in unhelpful ways through their own anxiety, reassurance and accommodation to the illness. Carers are introduced to high level communication and behaviour change strategies so that they can optimise their secure, age appropriate listening family base. By modelling changes in their behavior, not only do they show commitment, but this forms a context for vicarious learning of self‐management skills. At stage 3, guiding individual recovery, they are taught compassionate communication skills attuned to the complex eating disorder emotions and motivational interviewing skills to help elicit change talk and planning. These support skills facilitate connection and change and can be taught using various media, including a carer and therapist manual (Langley et al., 2018; Treasure et al., 2009) and videos (Cardi et al., 2015) and through diverse formats including workshops, guided online/phone and group and individual/group support from trained carers with lived experience.

### 3.1 Possible benefits of family inclusive interventions

#### 3.1.1 Resource use outcomes—systemic family therapy and family‐based therapy

The previously described challenges presented by the complex impact of AN on the individual and the social network mean that a discussion of cost effectiveness ideally should consider not just the individual but the family too. In this discussion of evidence to date, we use bed use (e.g. number of days in hospital) as one proxy marker of costs, as bed costs heavily dominate inpatient costs. However, it is often difficult to determine bed days due to discrepancies in studies categorising this as patient ‘drop out’ rather than a form of ‘rescue treatment’ (Albano et al., 2019; Gregertsen et al., 2019). Additionally, we include time spent caregiving as a proxy measure of carer costs where this is available.

As exemplified in the NICE guidelines, decision making for planning services is guided in part by cost effectiveness. However, this is not the only driver of evidence‐based practice. Patient/care giver preference and clinical knowledge are also of relevance. However, there is a paucity of these strands of evidence in eating disorders. In the following section, we aim to consolidate what is known by extracting the highest level of evidence from systematic reviews and meta‐analysis of cost effectiveness, clinical moderators and patient and carer feedback where possible.

The literature base for the evidence of family‐based therapy for early stage illness in adolescent AN is the longest and largest. Nevertheless, a recently updated Cochrane review (Fisher et al., 2020) concluded that the overall strength of the evidence was low to moderate. Most studies have focused on patient body mass index (BMI) at the end of treatment as the primary outcome, with less information about the rest of the family.

Despite the large literature base, few studies have utilised a spread of key outcomes, such as length of hospital stay (see Table 1). Studies that do refer to resources used during treatment are often qualitative, referring to feedback from families and professionals regarding costs of treatment (Grange & Gelman, 1998; Krautter & Lock, 2004).

**TABLE 1:** A summary of family‐based therapy studies that provide outcome data on bed use and extent of treatment

Author	Clinical sample	Comparison	Participants	Demographics	Main outcomes
Lock et al. (2016)	Adolescent inpatient	FBT versus SyFT	FBT: *n* = 78 SyFT: *n* = 80	Mean age: 15.4 (SD = 1.8) years, range: 12–18 years Gender: 89.2% female	Number of days in hospital: SyFT: 655 FBT: 369 Rehospitalised after first 5 weeks of treatment: SyFT: 6 patients (13 rehospitalisations) FBT: 2 patients (6 rehospitalisations)
Gusella et al. (2017)	Adolescent outpatient	PIC versus NST	PIC: *n* = 32 NST: *n* = 14	PIC (*M* = 13.07 years, SD = 1.89), (3 males, 29 females) NST (*M* = 14.36 years, SD = 1.37), (0 males, 14 females)	Required hospitalisation on a psychiatric unit for weight restoration NST: 71.4% PIC: 34.4% Number of days in hospital: NST: 50.0 days PIC: 19.1 days Required tube‐feeding: NST: 42.9% PIC 6.3%

Abbreviations: FBT, family‐based therapy; NST, non‐specific therapy; PIC, parents in charge; SD,  standard deviation; SyFT, systemic family therapy.

As shown in Table 1, in an adolescent inpatient context, FBT was associated with around half the number of days in hospital and around half the number of overall readmissions than systemic family therapy. It should be noted that in this study (Lock et al., 2016), around 1 in 10 participants were male, and so more work is needed to understand whether these findings are relevant to all genders. As shown in Table 1, the Parents in Charge (PIC) approach, a component of FBT, which sees clinicians empowering parents/carers to take charge of meals, when used in an adolescent outpatient context, was associated with the need for around 50% fewer hospital admissions, a shorter length of stay where an admission was required and a reduced use of nasogastric feeding than a non‐specific therapy which involved expert driven psychoeducational family sessions. This suggests that fewer resources are required when families are involved in treatment and although limited by the small number of studies with small sample sizes; these data may indicate that supporting parents to take control of meals may be useful, at least for younger adolescent patients.

There is some evidence that variation in baseline variables or the form or content of family treatment can moderate outcome. The first example was the finding that FBT was not superior to individual therapy for adults with a protracted illness or for people with adult onset of illness (Eisler et al., 1987; Russell et al., 1987). A further example is that different family presentations, such as high levels of maternal criticism, respond better to separated family therapy rather than conjoint (Eisler et al., 2007), suggesting FBT may not be appropriate for all family dynamics. Agras et al. (2014) found that Systemic Family Therapy resulted in significantly more weight gain for individuals who presented with greater obsessive‐compulsive psychopathology; however, those with lower scores gained a greater amount of weight with FBT, indicating that whilst FBT is an effective form of support, it is not ’one size fits all’.

### 3.2 Patient and carers' feedback on family‐based therapy

Some parents reported positive experiences with FBT as a structure to directly utilise their strengths and knowledge in their child's care (Parent & Parent, 2008). Individuals themselves also report that their parents' involvement was beneficial to their recovery (Chen et al., 2010). FBT has sometimes been promoted as a gold‐standard, fully effective intervention; the implication that follows is that any failure is due to those implementing the model that is the parents (Conti et al., 2017; Wufong et al., 2019). As a result some parents experienced guilt, anxiety and a sense of parenting failure if they were unable to achieve weight restoration in FBT (Conti et al., 2017; Wufong et al., 2019). The limited focus on refeeding in the early stage with elements of a wider formulation such as comorbidities and environmental exposures relegated to the third stage was problematic for some families, especially if challenging premorbid temperamental and developmental traits were present (Williams et al., 2020). Other families expressed concern that they were not taught how to manage their child's distress, and commented on the trauma this produced for the family (Conti et al., 2017).

Moreover, this intervention requires a significant amount of resources from the family, by asking them to engage in intensive treatment for large periods of time, potentially spanning years (Wufong et al., 2019). Whilst this aspect of the intervention has not been assessed adequately, many parents report having to leave employment to care for their child (Parent & Parent, 2008) or are fearful about losing their jobs (Williams et al., 2020). Not all families have the financial ability to commit to this level of care.

### 3.3 Resource use outcomes—the New Maudsley Model for collaborative care

A systematic review and meta‐analysis of self‐management approaches for carers of people with AN included two that used the NMCC (Albano et al., 2019). Drop out (including rescue treatment) was reduced, but clinical outcomes were similar. Bed use and carers' time from recent studies using NMCC are shown in Table 2. The intervention in both the adult and adolescent group produced a moderate sized reduction in time spent caring, and at the same time, bed use was reduced. Moreover there was a small to moderate improvement in the wellbeing of both carers and patients in the intervention group (Hodsoll et al., 2017; Hibbs et al., 2015; Magill et al., 2016). A similar pattern of less bed use and improved wellbeing was found using a hybrid intervention of NMCC and recovery MANTRA for patients (Adamson et al., 2019). Taken together these findings suggest that NMCC (a guided care management with moderate costs) can offer an intervention that can reduce service costs.

**TABLE 2:** A summary of New Maudsley Collaborative Care studies that have outcome data on bed use, readmissions and carer time spent caring

Author	Clinical Sample	Comparison	Participants	Family Outcome	Service Outcome
Hibbs et al. (2015)	Adult inpatient	TAU^a^ versus NMCC + TAU	NMCC: *n* = 86 Age median 23.16 (range 12.52–62.72) Gender: Female *n* = 83 Male *n* = 3 Carer: *n* = 134 Age median 52.22 (22.22–78.54) Gender: Female *n* = 80 Male *n* = 54	TSC at 6 months after discharge: NMCC + TAU: 22 (range = 0–478 h/month) TAU: 31.90 (range = 0–378 h/month) (*p* = 0.05; estimated group difference = 0.63 TSC at 12 months after discharge: NMCC + TAU: 17.50 (range = 0–466 hours/month) TAU: 20 (range = 0–379 h/month) (SC = 0.29) (p = 0.88); estimated group difference = 1.04	Length of stay: NMCC + TAU median = 148 (IQR = 28–991) TAU median = 163 (IQR = 33–570) Inpatient days (by 6 months): NMCC + TAU 927 days TAU: 1276 days Inpatient days (at 12 months): NMCC + TAU: 499 TAU: 1495 Readmission rate (at 1 year): NMCC + TAU: 27% TAU: 32%
Hodsoll et al. (2017)	Adolescent outpatient	TAU^a^ versus NMCC + TAU	TAU *n* = 50 TAU plus NMCC with guidance *n* = 50 NMCC without guidance TAU *n* = 49 Patients: Mean age 16.9 years (range: 13–21) 92% female Carers: Mean age = 48 years (SD = 5.2)	NMCC carers spent less time care giving (*D* = 0.4, *p* = 0.04) at one year	Rescue admission (1 year) 19% versus 28%
Adamson et al. (2019)	Adult inpatient	TAU^b^ versus NMCC + GSC + TAU	Patients *n* = 31 (all female) Mean age: 27.0 (8.8) Carers *n* = 21 (80% female)		WWG: NMCC + GSC + TAU: 0.51 kg TAU: 0.47 kg Mean LOS (SD): 88 (34) versus 112 (71)

*Note:* Estimated group difference is a coefficient estimating the treatment effect of NMCC + TAU minus TAU.

Abbreviations: *D*, Cohen's *D* effect size estimation; GSC, guided self care; IQR, Interquartile range; kg, kilograms; LOS, length of stay; NMCC, New Maudsley Collaborative Care; TAU , Treatment as usual; TSC, Time spent caring (hours per month); WWG , weekly weight gain.

^a^ TAU comprised of inpatient or day‐patient treatment.

^b^ TAU comprised of nutritional and medical interventions as well as individual and group psychotherapy.

### 3.4 Patient and carers' feedback on the New Maudsley Model for collaborative care

In one implementation of the NMCC, carers were asked to rate their experiences of the intervention and reported that it was easy to understand, the facilitators were knowledgeable, the intervention was of a high standard and they would be highly likely to recommend the intervention to others with mean scores from 4.18/5 to 4.91/5 (Pépin & King, 2013). Feedback elicited from patients whose caregivers had accessed the intervention shows a positive attitude to their caregivers' involvement, with feedback that they had acquired new skills and information, their relationship had improved, their caregiver had changed their approach to the illness and showed an increase in compassion towards them. Patients also reported that caregivers showed reduced anxiety and greater confidence and hope. In addition to this positive feedback, patients also raised concerns that they had to adjust to their caregivers new behaviours, that they were worried about possible blurring of boundaries between their caregiver and clinical team, and patients also expressed that their caregiver would need ongoing support and skills training to continue to support their recovery (Goddard, Macdonald, & Treasure, 2011).

### 3.5 Implementation, reach and spread of family‐based therapy and the New Maudsley Model for collaborative care

There has been widespread implementation of FBT in child and adolescent services. Interventions involving collaborative partnership working with parents and carers (NMCC) have also been disseminated (with a variety of accessible training materials) and implemented often through charities and voluntary organisations (BEAT, 2020; Rhind et al., 2014). The involvement of families is regarded as good practice within integrated care for adults with AN. There is however a gap in our knowledge about what works for whom and how and why and there is need to consider how to help families with features that portend a less good prognosis. Questions remain as to whether involving families more effectively at an early stage (Brown et al., 2018) can be an effective form of secondary prevention and whether a greater involvement of a wider net of social support is of greater value for later stages. Further, the evidence base largely encompasses studies conducted in the United States, Canada, Australia, United Kingdom and Western European countries, and it will be important in future work to investigate whether the same family based approach will have equal utility in other countries and settings and what adaptations might be useful to support families with EDs from a wider range of backgrounds.

## 4 CONCLUSIONS

The overall conclusion is that involving the family and/or wider forms of social support in the management of AN can be an effective strategy to augment professional help. Another benefit, although not widely measured in the literature, is reduced resources in the form of reduced bed use, with less need for nasogastric feeding, allowing families to avoid being separated with loved ones in hospital for extended periods. However, clinical judgment is needed to shape the optimal form of the intervention. For example, most of the evidence for FBT is for patients in the early stage of illness and involves patients with no or minimal comorbidities. It may be that those with premorbid comorbidity such as ASD or obsessive compulsive disorder may require a different approach. Also, the costs for the family/social support also need to be considered; even if this is freely given, especially for those with features signaling a poor prognosis, a mixed model of care, in which the family members are involved at a developmentally appropriate level (with the proviso that development may be profoundly delayed because of both physical and social factors), as an essential component of integrated care is needed.

## CONFLICT OF INTEREST

Janet Treasure receives publisher remuneration for Langley et al. (2018). *Caring for a Loved One with an Eating Disorder: The New Maudsley Skills‐Based Training Manual*. Routledge and Treasure, Smith, & Crane (2016). *Skills‐based caring for a loved one with an eating disorder: The new Maudsley method*. Routledge. Amy Harrison, Stacey Parker, and Oyenike Oyeleye declare that they do not have any conflicts of interest.
